# Synergistic modulation of signaling pathways to expand and maintain the bipotency of human hepatoblasts

**DOI:** 10.1186/s13287-019-1463-y

**Published:** 2019-12-02

**Authors:** Tingcai Pan, Yan Chen, Yuanqi Zhuang, Fan Yang, Yingying Xu, Jiawang Tao, Kai You, Ning Wang, Yuhang Wu, Xianhua Lin, Feima Wu, Yanli Liu, Yingrui Li, Guodong Wang, Yin-xiong Li

**Affiliations:** 1 Institute of Public Health, Guangzhou Institutes of Biomedicine and Health (GIBH), Chinese Academy of Sciences, Guangzhou, 510530 China; 2 University of Chinese Academy of Science, Beijing, 100049 China; 3 Key Laboratory of Regenerative Biology, South China Institute for Stem Cell Biology and Regenerative Medicine, Guangzhou Institutes of Biomedicine and Health, Chinese Academy of Sciences, Guangzhou, 510530 China; 4 Guangdong Provincial Key Laboratory of Biocomputing, Guangzhou Institutes of Biomedicine and Health, Chinese Academy of Sciences, Guangzhou, 510530 China; 5 The Second Affiliated Hospital, Guangzhou Medical College, Guangzhou, 510260 China; 6 iCarbonX(Shenzhen) Company Limited, Shenzhen, 518000 China; 7 The First Affiliated Hospital, Sun Yat-Sen University, Guangzhou, 510080 China; 8 Guangzhou Regenerative Medicine and Health Guangdong Laboratory, Guangzhou, 510005 China

**Keywords:** Human iPSCs, Hepatoblasts, Expansion, Bipotency maintenance, Self-renewal

## Abstract

**Background:**

The limited proliferative ability of hepatocytes is a major limitation to meet their demand for cell-based therapy, bio-artificial liver device, and drug tests. One strategy is to amplify cells at the hepatoblast (HB) stage. However, expansion of HBs with their bipotency preserved is challenging. Most HB expansion methods hardly maintain the bipotency and also lack functional confirmation.

**Methods:**

On the basis of analyzing and manipulating related signaling pathways during HB (derived from human induced pluripotent stem cells, iPSCs) differentiation and proliferation, we established a specific chemically defined cocktails to synergistically regulate the related signaling pathways that optimize the balance of HB proliferation ability and stemness maintenance, to expand the HBs and investigate their capacity for injured liver repopulation in immune-deficient mice.

**Results:**

We found that the proliferative ability progressively declines during HB differentiation process. Small molecule activation of Wnt or inhibition of TGF-β pathways promoted HB proliferation but diminished their bipotency, whereas activation of hedgehog (HH) signaling stimulated proliferation and sustained HB phenotypes. A cocktail synergistically regulating the BMP/WNT/TGF-β/HH pathways created a fine balance for expansion and maintenance of the bipotency of HBs. After purification, colony formation, and expansion for 20 passages, HBs retained their RNA profile integrity, normal karyotype, and ability to differentiate into mature hepatocytes and cholangiocytes. Moreover, upon transplantation into liver injured mice, the expanded HBs could engraft and differentiate into mature human hepatocytes and repopulate liver tissue with restoring hepatocyte mass.

**Conclusion:**

Our data contribute to the understanding of some signaling pathways for human HB proliferation in vitro. Simultaneous BMP/HGF induction, activation of Wnt and HH, and inhibition of TGF-β pathways created a reliable method for long-term stable large-scale expansion of HBs to obtain mature hepatocytes that may have substantial clinical applications.

**Graphical abstract:**

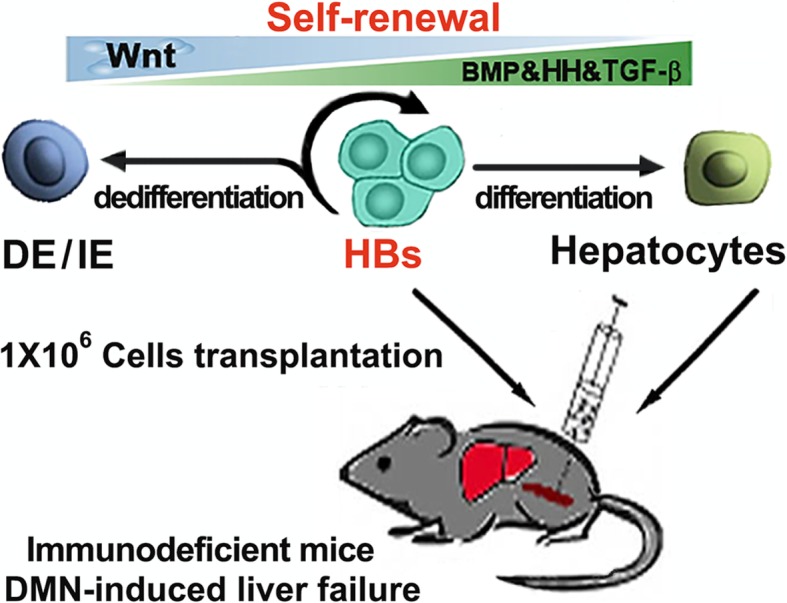

## Background

Hepatocyte transplantation and bio-artificial liver support have been clinically evaluated as effective methods for compensation of lost liver function and an alternative to liver transplantation [1–4]. However, these two methods are hindered by the shortage of viable organ donation and dysfunction of hepatocyte in vitro culture [5]. Thus, an alternative strategy to meet the demand for functional hepatocyte supply would represent a major clinical advance. Human pluripotent stem cell (hPSC)-derived hepatic lineage is considered to be a potentially good cell source for cell-based therapies and drug development process [6, 7]. The use of human embryonic stem cells (ESCs) in clinical trials is limited due to their low availability and ethical concerns regarding their use. However, ethical issues and intensive immunosuppressive treatment can be avoided by using patient-specific iPSCs [8]. Therefore, iPSC-derived hepatic cells are an ideal source for autologous cell-based therapy and extracorporeal artificial liver application.

Differentiation of hPSCs into hepatic cells has been achieved and improved during the last several years [9–11]. However, there are several limitations, which have not yet been addressed, especially related to their application in clinical trials and drug toxicology tests. The limitations include (a) heterogeneity of resulting cultures, as well as unpurified target cells; (b) low or ineffective hepatocyte-like functionality; (c) difficulty in obtaining large-scale hepatic cell populations. It is anticipated that therapeutic application would require 10^9^ graded hepatic cells per treatment [12, 13], while bio-artificial liver device application and drug discovery need even more. Thus, there is an urgent need to establish methods that can produce functional hepatic cells of high quality in large quantities.

In vitro culture of hepatocytes has low proliferative potential, while hepatoblasts (HBs) are capable of self-renewal and with potency to differentiate into hepatocytes and cholangiocytes. An ideal strategy would be to enable directed differentiation of human iPSCs to HBs with simultaneous cell expansion, which would enable up-scaling hepatic cell production. The expansion of HBs derived from hPSCs has been previously reported [14–18]. However, these studies relied mainly only on biomarker analyses to evaluate the bipotency and lacked many of the functional analyses and confirmation through animal transplantation was limited. In addition, some of them included undefined components such as fetal bovine serum or feeder cells leading to safety concerns for clinical applications [15, 17–19].

Recent studies identified small molecules which are used in the expansion of human HBs in defined chemical conditions [17, 20, 21]. However, these proliferative HBs were refractory to mature hepatocyte induction, which results in low ALB-positive cells and poor metabolic activity [14]. One possible explanation for the inability to derive functional hepatocytes is unstable maintenance of HB phenotype during expansion, which may be due to an ambiguous mechanism. Thus, a detailed understanding of the proliferation and differentiation mechanisms of human HB is important to establish a defined chemical method for expansion of human HB.

Here, we investigated the relationship of related signaling pathways with proliferation of human iPSC-derived HBs and established a chemically defined cocktail to synergistically regulate the related signaling pathways that optimized the balance of HB proliferation ability and stemness maintenance. The purified and expanded HBs stably retain phenotypes including the gene expression profile integrity and bipotency to differentiate into functional hepatocytes and cholangiocytes in vitro and in animal model. Therefore, our approach provides a defined chemical and serum-free method for long-term stable expansion of human iPSC-derived HBs for clinical applications.

## Methods

### Human iPSC culture and HB differentiation

Human iPSC lines (UC01 and UC15) and ESC line (H1) cultured in chemically defined mTeSR1 medium (Stem Cell Technologies) on a Matrigel matrix (Growth Factor Reduced, BD Bioscience). The human iPSC colonies were passaged using Accutase (life).

For the HB differentiation, we started the definitive endoderm (DE) stage induction first. Briefly, when human iPSCs reached nearly 70% confluence, mTeSR1 medium was replaced with differentiation medium (RPMI1640 [Gibco], supply with 1 × B27[minus insulin, Invitrogen]), containing 100 ng/mL Activin A (R&D Systems) and 3 μM CHIR99021 (CHIR) for 1 day, and on the following 2 days, CHIR was omitted from the medium. Then, DE population was cultured in differentiation medium, containing 20 ng/mL BMP2, 20 ng/mL BMP4, and 30 ng/mL FGF4 for 4 days to specify hepatic endoderm (HE), and then subsequently differentiated into HBs by treatment with differentiation medium containing 20 ng/mL BMP4 and 20 ng/mL HGF for 3 days. The medium was changed daily during the differentiation period. All grow factors were purchased from PeproTech except that indicate.

### HB proliferative culture

The differentiated day 10 cells were cultured on plates that pre-coated with Matrigel, and maintained in expansion basal medium (RPMI1640, 1 × B27 supplement, 1 × ITS [insulin-transferrin-sodium selenite, Sigma-Aldrich]) supplemented with growth factor and small molecule combination as indicated in Fig. 3a. Growth factors and small molecules were tested at appropriate concentrations as routinely used, including 20 ng/mL EGF, 20 ng/mL BMP4, 20 ng/mL HGF, 20 ng/mL FGF7, 3 μM CHIR, 5 μM A8301, 0.5 μM SAG, 10 μM Forskolin (FSK, Stemgent), 10 μM SB431542 (SB, Tocris), 0.5 μM Dorsomorphin (DM), and 0.5 μM Vismodegib (VM). Used small molecules were purchased from Selleck except indicated.

The EpCAM^+^/C-kit^−^ populations were sorted by cell flow cytometry, and single cell was seeded on Matrigel pre-coated 96-well plates and maintained in optimal AB_10_CEHS culture condition (expansion basal medium supplement with 5 μM A8301, 10 ng/mL BMP4, 3 μM CHIR, 20 ng/mL EGF, 20 ng/mL HGF, 0.5 μM SAG), for colony formation and other in vitro assays.

### Hepatocyte differentiation and bile duct induction

For hepatocyte differentiation, HBs were cultured in maturation medium hepatoZYME-SFM (Gibco), supplemented with 1X GlutaMAX, 10 ng/mL OncostatinM (OSM), 0.1 μM dexamethasone (DEX; Sigma-Aldrich), and 0.5 mM NH_4_Cl (Sigma-Aldrich) for 7 days. The medium was changed daily during the differentiation period.

For cholangiocyte differentiation in monolayer, HBs were cultured in basal medium (RPMI1640 [Gibco], supply with 1 × B27 [Invitrogen]), supplemented with 20 ng/mL EGF and 20 ng/mL HGF for 7 days. The medium was changed daily during the differentiation period.

For 3D bile duct induction, dissociated HBs were suspended in basal medium (RPMI1640 [Gibco], supply with 1 × B27 [Invitrogen]) supplemented with 20 ng/mL EGF and 20 ng/mL HGF, and mixed 1:1 with Matrigel. Then, the mixture was plated into 24-well plates (0.5 mL/well) and placed in an incubator at 37 °C for 2 h to allow the formation of 3D Matrix. The cells were cultured for approximately 1 week to allow the formation of bile duct-like structures. The medium was changed carefully every other day.

### Functional analyses of differentiated hepatocyte in vitro

For the albumin secretion assay, the culture media of 24 h incubated in differentiated cells were collected and evaluated using the human albumin ELISA Quantitation kit (Bethyl Laboratories) according to the manufacturer’s protocol. The results are representative of at least three independent experiments.

For the urea secretion assay, the culture media of 24 h incubated in differentiated cells were collected and stored at − 80 °C. Urea concentration was analyzed by LC/MS/MS API3000 and normalized with total cell protein concentration.

To evaluate the CYP450 activity, cultures were incubated with conventional probe substrates (CYP3A4: 6 μM midazolam, CYP2C9: 10 μM diclofenan, CYP2D6: 10 μM dextromethorphan) respectively, for quantifying metabolite production. After 2-h exposure, the culture medium was collected and stored at − 80 °C subsequently, and CYP450 activity was analyzed by LC/MS/MS API3000. Metabolite products were normalized to total cell protein.

To evaluate the glycogen production and storage ability, Periodic acid-Schiff (PAS) staining was performed. The cultured cells were fixed with 4% PFA for 30 min, and intracellular glycogen was stained using a PAS staining solution (Muto Pure Chemicals), according to the manufacturer’s instructions.

### Animal model and hepatic cell transplantation

Immune-deficient NOD-SCID-IL2RG^−/−^ mice (NSI mice, GIBH) were used as recipients of human hepatic cells. Before hepatic cell transplantation, 8-week-old NSI mice received DMN intraperitoneal injections (7 mg/kg, Sigma, 1.0% dissolved in saline) for 2 consecutive days for inducing acute liver injury. Two days later, 1 × 10^6^ hepatic cells were intrasplenic transplanted into the DMN-treated NSI mice. To monitor the transplantation state, recipient mouse blood and livers were harvested at different time points after hepatic cell transplantation. Additionally, human hepatocytes that were producing the ALB protein were identified in mouse liver by an antibody specifically recognizing human ALB. Serum and plasma were separated from mouse blood and stored at − 80 °C for liver function tests. All animal experiments were approved by the Animal Welfare Committee of GIBH. All protocols were approved by the relevant institutional animal care and use committee (IACUC).

### Statistical analysis

The data were analyzed with Sigma Plot 10.0 Statistical differences between two groups were tested with a two-tailed Student’s *t* test. Data is represented as mean ± SEM. Survival data were analyzed with the Kaplan-Meier test. For all tests, *p < 0.05 was considered significant.

More experimental details are described in Additional file 1. Information about the antibodies and primers used is in Additional file 1: Tables S1 and S2.

## Results

### Proliferative ability declined progressively during hepatic differentiation process

We adopted a stepwise differentiation protocol using serum-free chemically defined medium to induce HBs from human iPSCs based on previous protocols and reports on signaling regulation during embryonic hepatogenesis [11, 22]. Briefly, the protocol used Activin A and CHIR to induce definitive endoderm (DE) for 3 days, followed by BMP2, BMP4, and FGF4 to specify hepatic endoderm (HE) from day 4 to day 7. The HE cells were further differentiated to HBs by induction of BMP4 and HGF (B4H) from day 8 to day 10. Details are described in the experimental procedures.

**Fig. 1:**
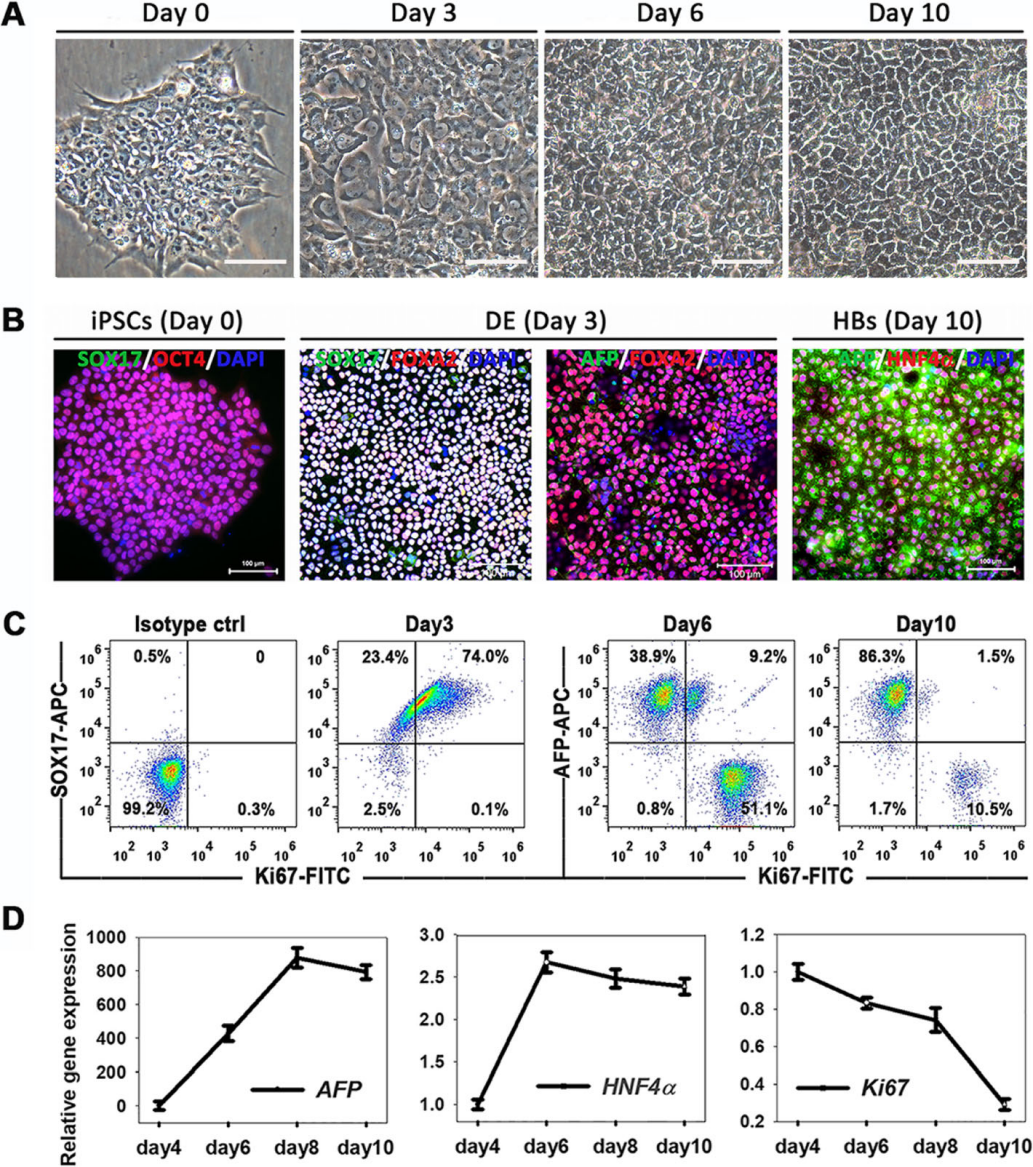
Proliferative ability declined progressively during HB differentiation process. **a** Sequential morphological changes in the differentiation of human iPSCs into HBs. Scale bars 100 μm. **b** Stage-specific protein expression during HB differentiation process. **c** Flow cytometric analysis for Ki67-positive cells during HB differentiation process. **d** *AFP*, *HNF4α*, and *Ki67* expression are analyzed by RT-PCR. Data are presented as mean ± SEM, *n* = 3

During the stepwise differentiation of HBs, sequential morphological changes and stage-specific protein analyses were performed at the end of each stage. At the DE stage (day 3), most cells (over 95%) were double-positive for DE-specific transcriptional factors, FOXA2 and SOX17. At day 10, nearly homogenous stages of the differentiated cells were observed and they co-expressed factors characteristic of HBs, AFP, and HNF4α (Fig. 1a, b), suggesting an effective sequential induction of iPSCs into DE and HBs. Importantly, we found that the proliferative abilities of these cells progressively declined during the differentiation process. Flow cytometry analyses showed that more Ki67 (a proliferative marker)-positive cells (74.1% at day 3 and 60.3% at day 6) were observed at early stages. These Ki67-positive cells decreased in the HB stage (12% at day 10), which was consistent with the subdued cell growth during the differentiation process (Fig. 1c). On the other hand, the expression of *Ki67* gradually declined before HE stage and dropped quickly thereafter, while hepatic genes *AFP* and *HNF4α* gradually upregulated as expected during hepatic differentiation. This confirmed the poor proliferative capacity of HBs that were cultured in the differentiation medium (Fig. 1d).

This observation suggests that (a) the combination of BMP/FGF/HGF typically used for HB induction was not enough to maintain the proliferative ability and (b) multiple endogenous signaling pathways synergistically coordinate the differentiation, proliferation, and stemness of HB. Clarifying the role of these signaling mechanisms may aid in intensive proliferation of HBs and maintain their bipotency.

### Signaling mechanisms synergistically regulate hepatic specification and proliferation

**Fig. 2:**
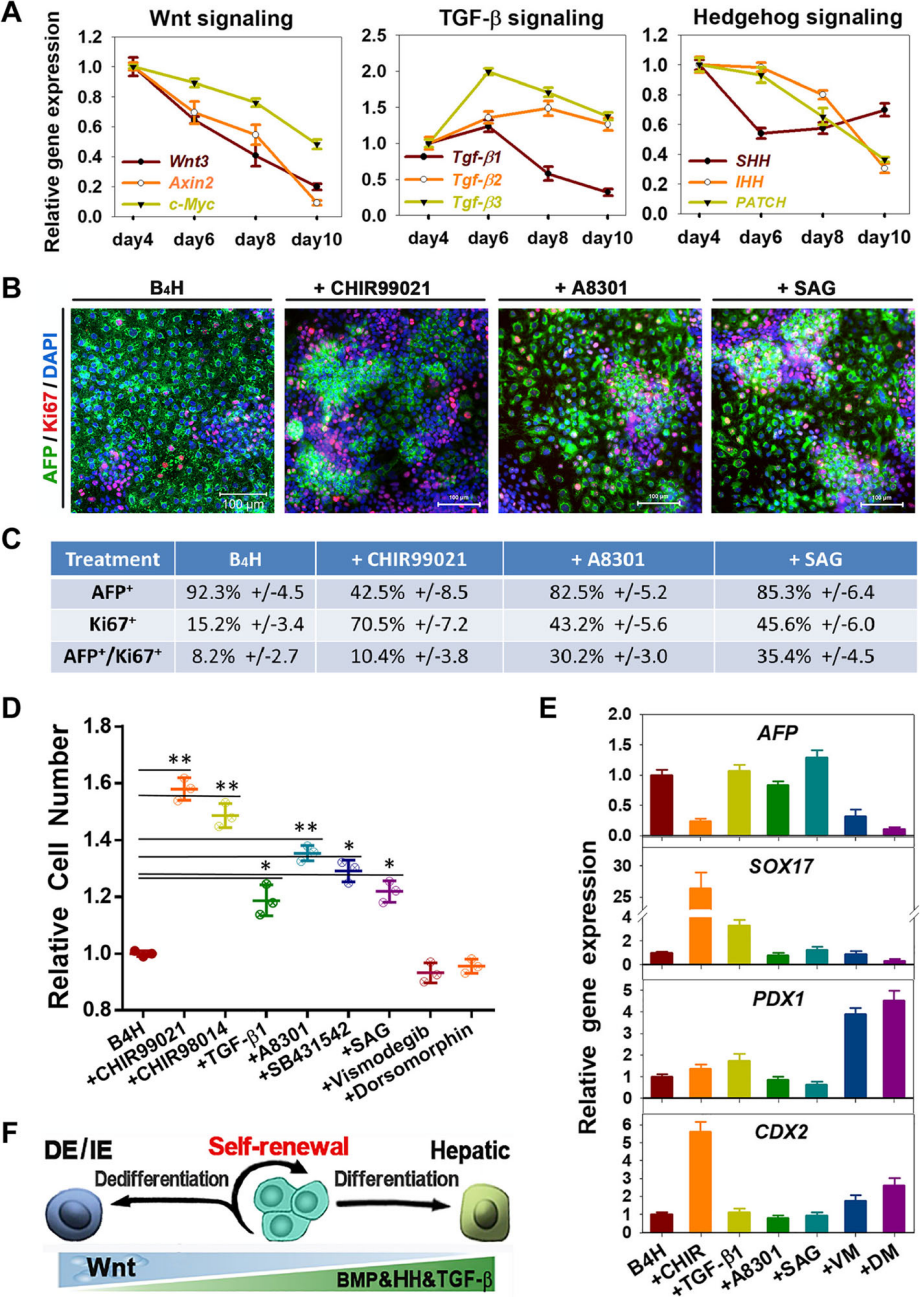
Synergistic regulation of signaling pathways for hepatic specification and proliferation. **a** Wnt, TGF-β, and HH signaling pathway-related gene expression was analyzed by RT-PCR. Data are presented as mean ± SEM, *n* = 3. **b** Immunostaining analyses of Ki67 and AFP expression after different small molecule treatment. **c** Efficiency of Ki67 and AFP expression after small molecule treatment, determined by counting positive cells. Efficiencies are presented as the percentage of positive cells plus or minus the SD of all fields counted. **d** Analyses of small molecule effect on HB proliferation. Data are presented as mean ± SEM, *n* = 3. *P < 0.05, **P < 0.01. **e** Transcript markers of different cell lineages in HBs treated with different small molecules. Data are presented as mean ± SEM, *n* = 3. **f** Model of signaling pathways that regulate self-renewal and differentiation of human HBs

In order to illustrate the status of signaling pathways involved in hepatic differentiation and cell proliferation, gene expression of members of Wnt, TGF-β, and hedgehog (HH) pathway were dynamically traced during the DE differentiation to HB stage. The expression of *Wnt3* and its downstream genes *Axin2* and *c-Myc* declined sharply, indicating that Wnt signaling was downregulated. For the expression of the three TGF-β ligand genes (*Tgf-β1*, *Tgf-β2*, and *Tgf-β3*), all three of them were increased from day 4 to day 6. From day 6 to day 10, expecting the expression of *Tgf-β1* was decreased, however, the expressions of *Tgf-β2* and *Tgf-β3* were still maintained at certain levels that may cause cell proliferation inhibition. The expression of HH signaling pathway genes *IHH* and *PATCH* dramatically decreased from day 6 to day 10, indicating that HH signaling declined from the HE to the HB stage (Fig. 2a). Meanwhile, *SHH* showed a slight increase from day 6 to day 10, probably due to BMP2 was removed in day 8 resulting in a decreased BMP signaling, which is an antagonist for SHH through Smads1/5/8 [23, 24]. This change may lead to the contrast changes between SHH and IHH through HH non-canonical pathway.

Recent studies have demonstrated that Wnt/β-catenin pathway support self-renewal of HBs and HH signaling is involved with hepatic progenitor cell proliferation [25, 26], whereas TGF-β signaling induces cell growth arrest. Thus, to test whether promoting Wnt and HH signaling or inhibition of TGF-β signaling improves the proliferative ability of HBs, therefore, agonist or antagonist small molecules were administrated for targeting these signaling pathways. Since *Ki67* (proliferative maker) expression reached to the lowest level, therefore, we used the cells in day 10 for the proliferation measurement for following 3 days. Cells were re-plated at the same density and cultured for an additional 3 days with the basic B4H medium with different agonist or antagonist administration respectively. Treatment with CHIR99021, a GSK-3β inhibitor that acts as a Wnt agonist, or TGF-β signaling inhibitor A8301 or HH signaling Smoothened activator SAG enhanced *Ki67* expression with 70.5%, 43.2%, and 45.6% respectively (Fig. 2b, c). However, co-staining with AFP indicated Ki67-positive cells had a weak or negative AFP expression in the CHIR group. While in the A8301 and SAG groups, most Ki67-positive cells maintained the AFP expression levels (Fig. 2c). Further, cell growth analyses confirmed that GSK-3β inhibitor (CHIR99021 or CHIR98014) or TGF-β signaling inhibitor (A8301 or SB431542) significantly improved cell growth. Treatment with SAG also increased the proliferation of HBs, but the proliferation was less than that achieved by CHIR treatment. Interestingly, TGF-β1 treatment also improved the proliferation slightly. In contrast, inhibition of BMP signaling by Dorsomorphin (DM) or HH signaling by Vismodegib (VM) fully blocks HB proliferation (Fig. 2d). These results demonstrated that GSK-3β inhibitors significantly promoted HB proliferation but diminished the HB phenotype, while TGF-β signaling inhibition and HH signaling activation stimulated HB proliferation and also sustained the characteristics of HBs.

To define the cell fate of these HBs after treatment with signaling inhibitors, gene expression of key biomarkers was analyzed. CHIR treatment dramatically diminished the *AFP* expression and increased the expression of *SOX17* (DE) and *CDX2* (intestinal endoderm), suggesting a phenotype shift from HB to intestinal endoderm. The treatment with TGF-β1 increased *AFP* expression slightly, but also induced the expression of *SOX17* and *PDX1* (pancreatic). However, A8301 could block the expression of *SOX17* and *PDX1* without a significant change in *AFP* expression. SAG promoted *AFP* expression the most among these molecules. Contrarily, VM or DM not only diminished *AFP* expression but also induced *PDX1* expression (Fig. 2e), suggesting HH signaling inhibition or BMP signaling inhibition may induced a cell faith shift from hepatic to pancreatic lineage.

In summary, activation of Wnt and HH signaling (CHIR and SAG) and inhibition of TGF-β signaling (A8301) promote HB proliferation. However, CHIR alone damages the HB characteristics due to dedifferentiation into endoderm or intestinal endoderm, while BMP and HH signaling are essential for preserving the HB phenotype (Fig. 2f). Thus, we speculated that proliferation or expansion resulted in HB phenotype loss, which could be avoided by synergistically regulating the BMP/WNT/TGF-β/HH signaling.

### Optimization of the HB expanding culture conditions

**Fig. 3:**
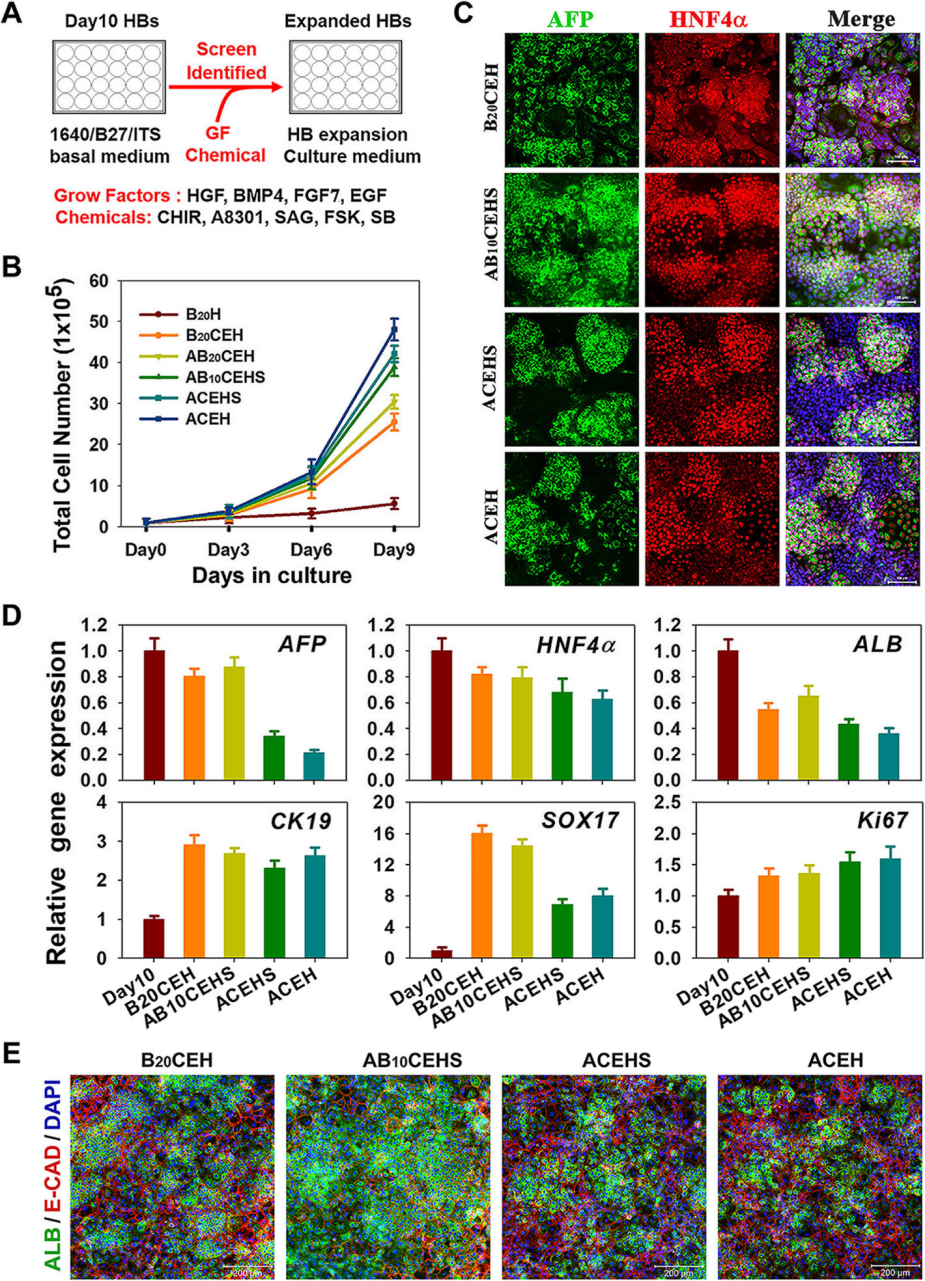
Optimizing the HB expanding culture condition. **a** Schematic of the strategy to identify culture conditions that sustain HB expansion. **b** Growth curve of HBs cultured in different conditions. Cell growth curves were analyzed by obtaining a cell count. Data are presented as mean ± SEM, *n* = 3. A: A8301; B_20_: 20 ng/mL BMP4; B_10_: 10 ng/mL BMP4; C: CHIR99021; E: EGF; H: HGF; S: SAG. **c** AFP and HNF4α-positive cells were examined. **d** Quantitative RT-PCR results showing expression of hepatic genes and *Ki67* in expanding HBs. Data are presented as mean ± SEM, *n* = 3. **e** Expressions of ALB and E-CAD were measured in the hepatocytes differentiated from HBs expanded with different conditions

In order to establish an optimized condition that promotes the expansion of HBs without diminishing their hepatic characteristics, as observed in our results and reported elsewhere [14, 17, 20], we screened for synergistically regulated signaling transductions (Fig. 3a). Cell growth curves of HBs were measured during a 9-day culture period (Fig. 3b). The basic BMP4 and HGF (B_20_H) cocktail had minimal proliferation activity, and the majority of cells lost their epithelial shape shifting to a fibroblastic shape (Additional file 1: Figure S1). Addition of CHIR and EGF (B_20_CEH) could endow HBs with minor proliferative activity; however, the expression of AFP and HNF4α decreased as observed earlier (Fig. 3c, first row). Further addition of A8301 (AB_20_CEH) resulted in increased proliferative ability of HBs and improved the expression of AFP and HNF4α, simultaneously. BMP4 concentration of 10 ng/mL and addition of SAG (AB_10_CEHS) further increased the expression of AFP and HNF4α (Fig. 3c, second row). On the other hand, withdrawal of BMP4 (ACEHS and ACEH) further promoted proliferation while led to decreased AFP and HNF4α expression, indicating the critical role of BMP signaling in HB stemness maintenance (Fig. 3c, third and fourth rows). Gene expression analyses further confirmed results that the optimal cocktail (AB_10_CEHS) increased the transcript level of *Ki67* and maintained the expression of HB marker genes, including *AFP*, *HNF4α*, and *CK19* (Fig. 3d).

Further, hepatocyte induction was performed to analyze the differentiation capacity of HBs, which were expanded in different conditions. Immunostaining revealed that ALB and E-CAD expression was the highest in hepatocyte derived from AB_10_CEHS cocktail (Fig. 3e). Finally, we concluded that the optimal culture cocktail for HB expansion was the AB_10_CEHS cocktail, which increased the cell number by approximately 40 times during the 9-day culture period and maintained the hepatoblast phenotypes simultaneously.

### Purification, single-cell colony, and long-term culture for HB expansion

**Fig. 4:**
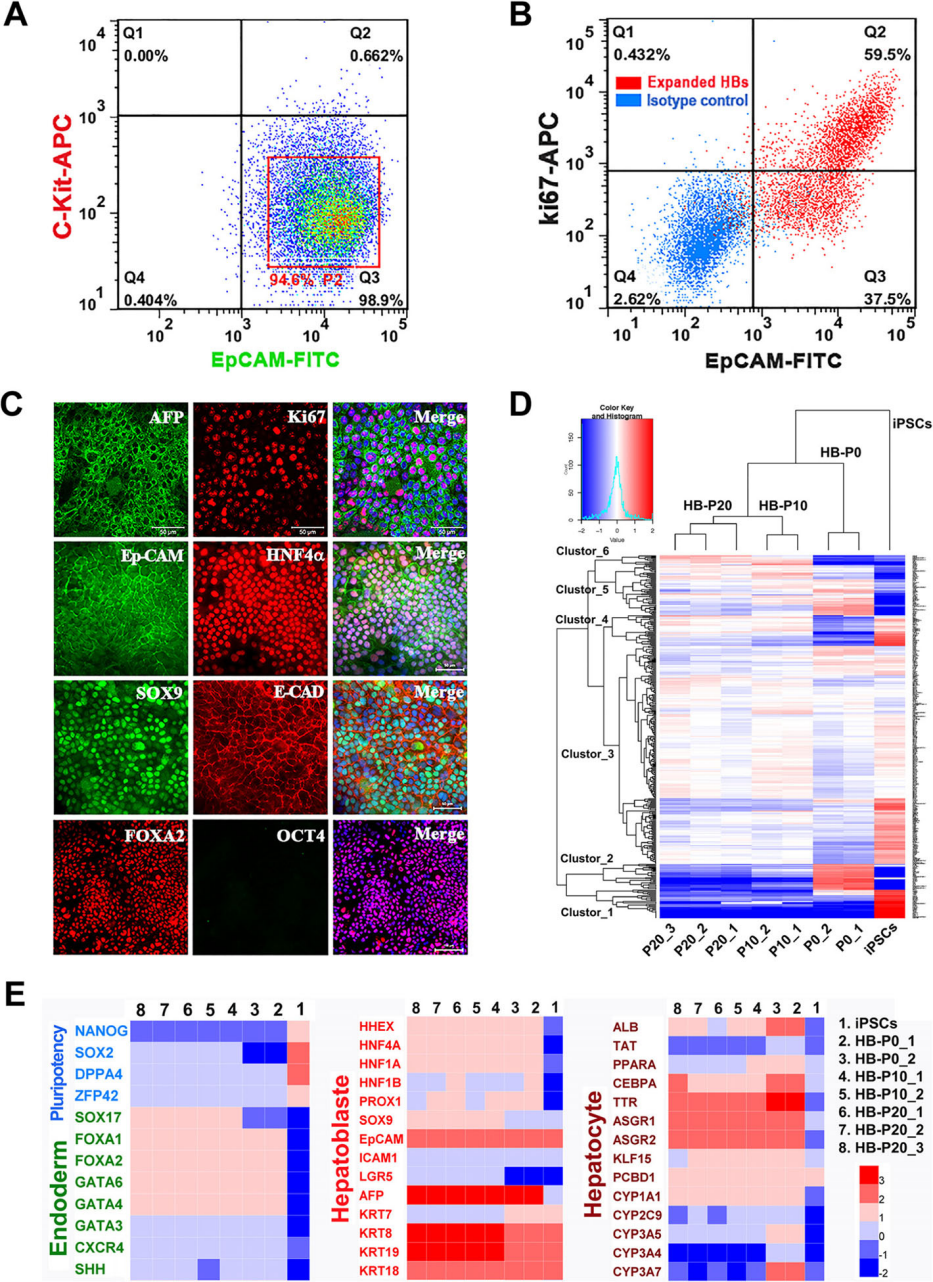
Purification, single-cell colony, and long-term culture for HB expansion. **a** Flow cytometric sorting of EpCAM^+^/C-kit^−^ HBs at day 10. **b** Flow cytometric analyses of EpCAM and Ki67 expression in expanded HBs (passage 10). **c** Immunostaining analyses of HBs after 10 passage expansion. **d** Hierarchical clustering analyses of mRNA profiling of expanded HBs (HB-P10 and HB-P20), unexpanded HBs (HB-P0), and iPSCs. **e** Heat map comparison of the gene expression profiles endodermal, HB, and hepatocyte among expanded and unexpanded different cell clone lines. Representative genes were indicated. Normalized fluorescent intensity values range from red (high) to blue (low) coloring

We next addressed whether HBs could undergo clonal expansion for long-term culture without diminishing their bipotency. We first used fluorescence-activated cell sorting (FACS) to sort EpCAM-positive and C-kit-negative iPSC-derived HBs (Fig. 4a). And colony formation assays showed that the EpCAM^+^/C-kit^−^ single cells were able to proliferate into colonies in AB_10_CEHS culture condition, and colonies were successfully expanded by serial passages (data not showed). Furthermore, the purified HBs underwent long-term culture for at least 20 passages, without significant morphologic change and obvious decrease in their proliferative ability. During expansion, these cells could be harvested, frozen, and thawed repeatedly.

For HB characterization, flow cytometry results showed that nearly 60% EpCAM-positive HBs (at passage 10) co-expressed Ki67 (Fig. 4b). Immunostaining further confirmed that a considerable number of AFP-positive cells co-expressed Ki67 (Fig. 4c), indicating preferable proliferative ability of HBs. Moreover, the expanded HBs also expressed HB biomarkers like HNF4α, SOX9, and FOXA2, but negative for pluripotent OCT4 (Fig. 4c).

Furthermore, transcriptome comparison between early (HB-P10, HB of passage 10) and late (HB-P20, HB of passage 20) passages of the expanded HBs demonstrated a similar gene expression profile, which resembled that of unexpanded HBs (HB-P0), although some differential expression patterns were observed (Fig. 4d). The heat map and mean expression profile graphs showed that both expanded and unexpanded HBs barely expressed pluripotency genes such as *NANOG* and *DPPA4*, which were highly expressed in control iPSCs (cluster 1). Notably, the unexpanded HBs expressed mature hepatocyte transcripts, including *ALB*, APO lipoproteins (*APOC1*, *APOC3*, and *APOE*), and other proteins involved in important hepatic detoxification functions (e.g., *GSTA1*, *GSTA2*). Upon expansion, some of these gene expressions were lost (cluster 2). Furthermore, cell proliferation-related transcripts (*Ki67* and *LIN28A*) were highly expressed in expanded HBs and iPSCs, but not in unexpanded HB samples (cluster 4). Other HB genes (*CK8*, *CK18*, and *CK19*) were expressed in both expanded and unexpanded HBs but not in iPSCs (cluster 5). A detailed analysis of genes related to HB bipotency, hepatic lineage, and pluripotency was performed to confirm the gene profile integrity upon expansion (Fig. 4e). Additionally, the expanded HBs also retained normal karyotypes after long-term culture (10 passages) (Additional file 1: Figure S2). Altogether, these genome-wide gene expression results indicated that both early and late passages expanded HBs had a similar expression profile to unexpanded HBs.

To further confirm the HB differentiation and expansion methodology, we tested two more hPSC lines (iPSC line UC15 and ESC line H1), using the optimized conditions that previously applied to the iPSC line UC01. Results showed that these two cell lines also could sequentially differentiate into DE cells and HBs, with high efficiency similarity as UC01 cells and expressed stage-specific markers (Additional file 1: Figure S3). More importantly, the AB_10_CEHS culture condition also could expand these HB derived from UC15 iPSCs and H1 ESCs for a long-time culture, and there were no significant differences among their proliferative abilities (Additional file 1: Figure S4A). Further immunostaining analyses showed that the majority of expanded HBs (P10) co-expressed AFP and Ki67 (Additional file 1: Figure S4B) and other HB biomarkers (Additional file 1: Figure S4C). It indicated this AB_10_CEHS expansion formula can be duplicated in other hPSC lines.

### Differentiation of expanded HBs into functional hepatocytes and biliary lineages in vitro

**Fig. 5:**
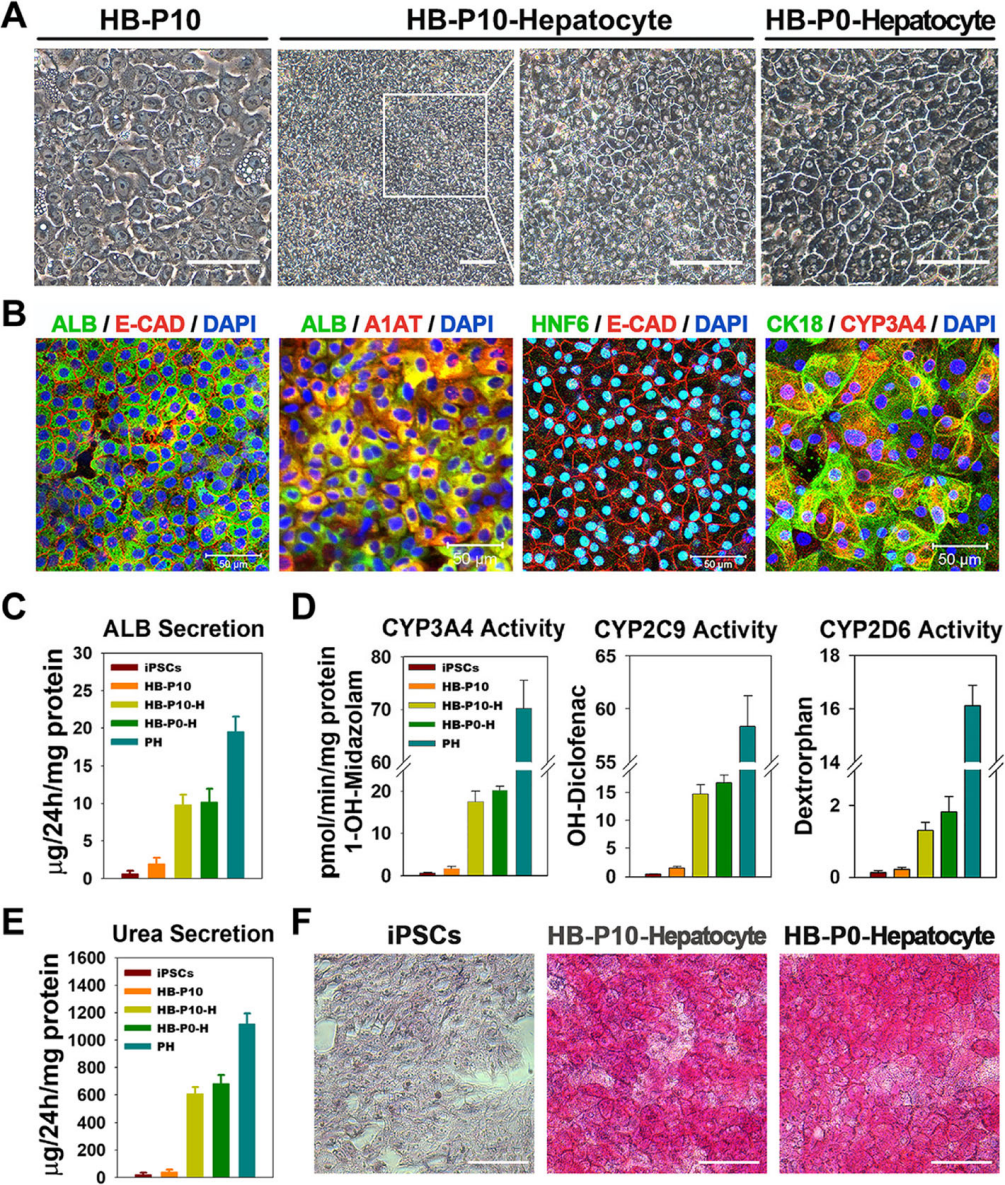
Differentiation of expanded HBs into functional hepatocytes in vitro*.* **a** Phase-contrast images of differentiating cells. Scale bar 100 μm. **b** Expanded HB-derived HLCs (HB-P10-Hepatocyte) expressed mature hepatocyte-specific markers. **c** ALB secretion was analyzed. Data are presented as mean ± SEM, *n* = 3. **d** CYP450 activity assay of different origin of hepatocytes. Data are presented as mean ± SEM, *n* = 3. **e** Urea secretion among different origin of hepatocytes. Data are presented as mean ± SEM, *n* = 3. **f** Periodic acid-Schiff (PAS) staining on different origin of hepatocytes. Scale bar represents 100 μm

We further investigated whether long-term culture expanded HBs retain bipotency to differentiate into both hepatocytes and biliary lineages. Firstly, cells were differentiated into hepatocyte-like cells (HLCs). The expanded HBs (HB-P10) were harvested from AB_10_CEHS expansion medium and then cultured in condition for hepatic maturation. After 7 days, these expanded HBs derived hepatocytes (HB-P10-Hepatocyte or HB-P10-H) displayed a homogenous and typical mature hepatocyte-like polygonal morphology, replicating the morphological features of the unexpanded HB-derived HLCs (HB-P0-Hepatocyte or HB-P0-H) (Fig. 5a). Immunostaining analyses showed that there was no significant difference in yield of HLCs between the expanded and unexpanded HBs, as evidenced by co-staining of a range of mature hepatocyte-specific proteins, including ALB, A1AT, HNF6, CK18, and CYP3A4 (Fig. 5b).

Further analyses were performed to examine typical functional activities of mature hepatocyte. HLCs showed a similar ALB secretion pattern between the expanded (HB-P10-H) and unexpanded (HB-P0-H) groups, which correspond to approximately 50% of ALB secretion of the human primary hepatocyte (PH) (Fig. 5c). Next, we assessed the detoxification functions of the HLCs by characterizing the activities of cytochrome P450 (CYP) enzymes. Three CYP isoforms were tested by measuring the increase in CYP isoform metabolites in response to exposure to their respective probe substrates. There was no statistically significant difference in all three CYP isoforms’ activity between expanded and unexpanded HB-derived HLCs (Fig. 5d). The measurement of urea secretion indicated that both HLC groups were similar, about one third the secretion by the PH (Fig. 5e). Periodic acid-Schiff staining (PAS) showed that both HLC groups displayed similar cytoplasmic glycogen storage ability (Fig. 5f). Undifferentiated iPSC was used as a negative control. For confirmation purposes, the expanded HB derived from two more hPSC lines (iPSC UC15 and ESC H1) were parallel tested as the UC01 for hepatocyte differentiation, and similar maturation and functional results were observed (Additional file 1: Figure S5).

Next, we investigated whether HBs can differentiate into cholangiocyte-like cells and form bile duct-like structure. After 7 days of cholangiocyte induction in monolayer culture, CK19-positive cells were observed, although ALB-positive HLCs were also observed in the culture (Additional file 1: Figure S6A, left). After approximately 1 week in 3D culture, HBs formed bile duct-like structures. These bile duct-like structures demonstrated epithelial polarity with CK7 and CK19 on the basolateral region and F-actin at the apical region (Additional file 1: Figure S6A, Right). Similar results were also obtained on two other expanded HBs derived from UC15 or H1 (Additional file 1: Figure S6B). Taken together, these results indicated that expanded HBs retained bipotency after long-term expansion, with the ability to differentiate into both hepatocytes and biliary lineages in vitro.

### Transplantation of expanded HBs rescued acute liver failure

**Fig. 6:**
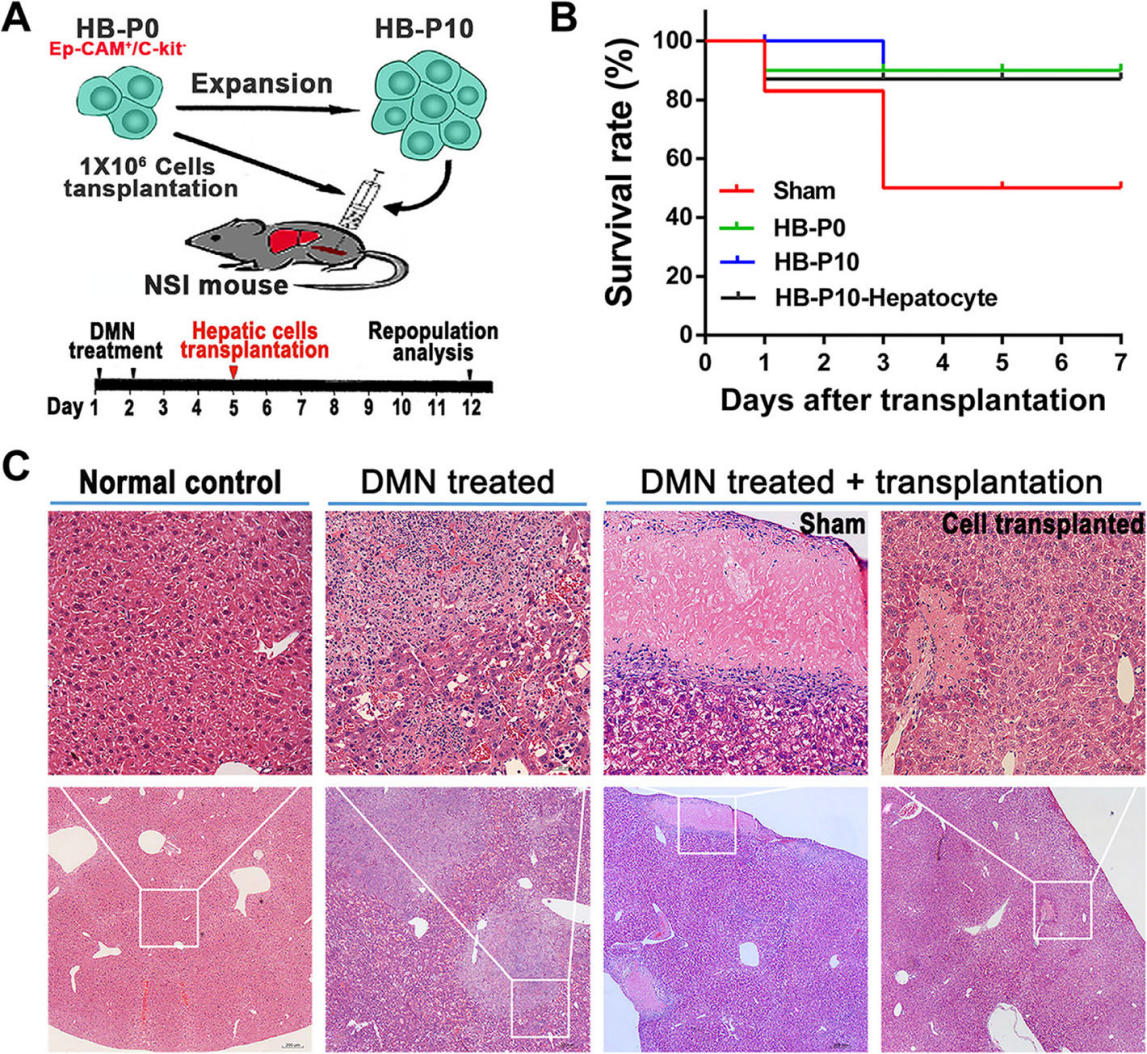
Transplantation of expanded HBs rescued acute liver failure. **a** Schematic diagram depicting immune-deficiency mouse liver injury model and hepatic cell transplantation experimental schedule. **b** Survival curve of mice. **c** Hematoxylin and eosin staining in mouse liver

To determine whether the expanded HBs could mature into functional hepatocytes in vivo, we transplanted HBs or HB-derived HLCs into DMN-induced acute liver failure of NSI mice (Fig. 6a). The Kaplan-Meier survival estimates were determined for 7 days after cell transplantation. In the sham control group, death caused by acute liver failure occurred as early as 3 days and nearly half (6 of 12) died within 7 days after the DMN injection. For the experimental groups (*n* = 12 per group), survival rates of all three groups (unexpanded HBs (HB-P0), expanded HBs (HB-P10), or expanded HB-derived HLCs (HB-P10-Hepatocyte)) were over 85% throughout the examination period (Fig. 6b).

Hematoxylin and eosin (H&E) staining displayed massive necrosis loci associated with inflammatory cell infiltration in the liver, which was noted 2 days after DMN-induced acute liver failure (Fig. 6c, second panel). After 1 week of hepatic cell transplantation, the necrosis loci dramatically decreased and morphologically restored the recipient liver tissues. In contrast, in the sham control liver, the necrosis loci were still widespread (Fig. 6c, right).

### Repopulation of mouse injured liver by transplanted HBs

**Fig. 7:**
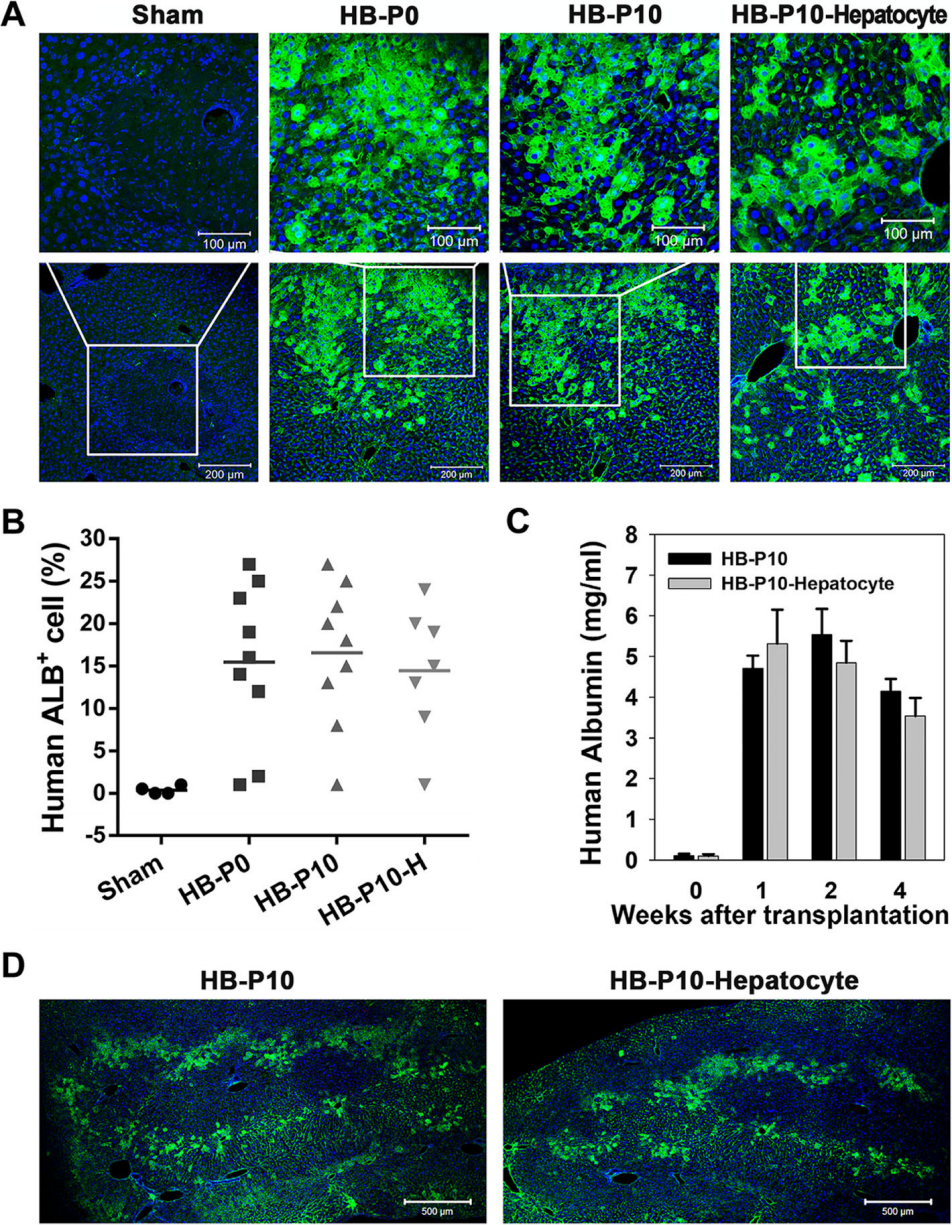
Repopulation of mouse injured liver by transplanted HBs. **a** Engraftment of transplanted human hepatic cells (human ALB, green) in mouse liver after 1 week transplantation. **b** Percentages of engrafted cells in recipient mouse liver sections 1 week after transplantation. **c** Human ALB secretion in the mouse serum. Data are presented as mean ± SEM, *n* = 4. **d** Repopulation with transplanted cells in mouse liver 4 weeks after transplantation

To analyze repopulation efficiency, immunostaining with human ALB antibody was conducted 1 week after transplantation. Representative patterns of positive staining of human ALB in the HBs and HB-derived hepatocytes transplanted liver were showed, indicating that both unexpanded (HB-P0) and expanded HBs (HB-P10) could differentiate into mature hepatocyte in vivo. Expanded HB-derived HLCs (HB-P10-Hepatocyte) also could engraft in the recipient liver (Fig. 7a). The overall results for experimental groups showed similar percentage of engrafted cells in recipient mouse liver sections and that human ALB was detected in 21 out of 25 mice receiving hepatic cells (Fig. 7b). Examination of human ALB in the mice’s serum after hepatic cell transplantation showed similar human ALB secretion in HB-P10 and HB-P10-Hepatocyte transplanted groups (Fig. 7c). Four weeks after transplantation, human ALB-positive cells were observed both in expanded HBs and hepatocytes transplanted mouse liver (Fig. 7d), which was consistent with the human ALB secretion data.

No teratomas or other tumor types were found in any of the transplanted recipients during a 4-week time frame. Therefore, we concluded that purified and long-term expanded HBs could engraft and differentiate into hepatocytes after transplantation.

## Discussion

HB proliferation is an integral process in embryogenesis, and it is observed that the cell proliferation ability gradually declines during HB induction in vitro. Thus, it is difficult to obtain sufficient population of HBs in direct hepatic differentiation protocols, limiting their large-scale production to meet the clinical demands. Therefore, there is an urgent need to establish efficient method for generating abundant and homogeneous HBs.

In this study, we demonstrated that CHIR could improve the HB growth significantly, which was in line with recent reports [14, 25, 27]. However, CHIR treatment led to loss of HB identity and reversing the gene expression pattern from hepatic to an intestinal endoderm pattern. This result is consistent with previous studies, which showed that Wnt/beta-catenin signaling acts directly on endoderm to induce CDX2, and direct intestinal induction [28, 29]. Another recent study proposed that CHIR increases the transcription of hepatic genes at the HB stage, which was contrary to our results. This may be attributed to synergistic action of TGF-β1 and HGF used in the previous study [25]. In addition, another recent study reported that inhibition of Wnt signaling using WIF-1 and DKK-1 facilitated the acquisition of hepatic lineage [9]. These findings suggest a dual role of Wnt signaling in HB differentiation and expansion.

We also demonstrated that simultaneous inhibition of TGF-β and activation of HH signaling could improve self-renewal, proliferation, and bipotency maintenance of HBs. This result is consistent with previous reports that inhibition of TGF-β signaling could maintain phenotype of HBs by inhibiting cell differentiation towards cholangiocytes [30]. Other reports demonstrated that TGF-β signaling regulate the differentiation of HBs towards bile ductal cells in liver development and regeneration [31, 32]. HH signaling plays an inhibitory role for the pancreas in the early developmental stage. On the other hand, it promotes the proliferation and differentiation of murine and human hepatic progenitors [26, 33, 34]. It is noteworthy that BMP signaling also plays a vital role in bipotency maintenance and proliferation of HBs. In the absence of BMP signaling, the differentiation shifts to other lineages [35].

Considering the overall profile of hepatic (*AFP*), pancreatic (*PDX1*), and intestinal (*CDX2*) markers, we assume that expanding HBs require cross-talk between signaling pathways and sequential balance to sustain its bipotency. We treated HBs with CHIR and A8301 to stimulate its proliferation and simultaneously integrated BMP/HH signals during expansion to block pancreatic and intestinal endoderm differentiation, which resulted in hepatic specification. After screening multiple cocktail combinations, we established that AB_10_CEHS cocktail could stably support the expansion of HBs by self-renewal, without diminishing their bipotency. The expanded HBs retained the ability to differentiate into both functional hepatocytes and biliary lineages. The HB-derived HLCs express CYP3A4 and other hepatocyte-specific proteins. Moreover, the CYP enzyme metabolic activity of the HLCs were comparable to freshly isolated PH, suggesting a valuable alternative cell resource of these cells in artificial liver support system and drug toxicology analyses.

So far, the repopulation capacity of transplanted hepatocyte has been limited. Previous study reported that transplanted HBs was able to repopulate the liver and improve liver function [36]. A recent study even demonstrated that fetal liver progenitor cells could replace liver mass to a greater extent than hepatocytes [37]. In addition, another recent study reported that transplanted mouse hepatic progenitor cells of biliary origin demonstrated bi-lineage differentiation into hepatocytes and cholangiocytes, causing significant structural and functional improvement of the mouse injured liver [38]. In our present study, HBs transplanted into NSI mice with acute liver failure could engraft and differentiate into hepatocytes, which restored hepatocyte mass. This led to considerable liver repopulation and successfully bridged the acute liver failure recipients through the critical period for survival.

## Conclusions

In summary, our experimental setting in which small molecules mediated exogenous modulation of signaling pathways is sufficient for the expansion of HBs derived from human iPSCs. This study provides strong in vitro evidence supporting the possibility for HB expansion without affecting its bipotency. Although more detailed analyses and more stringent validations are required, our study provides an ideal in vitro platform for investigating the mechanism of human HB expansion. Furthermore, this chemically defined and serum-free hepatic differentiation and expansion cocktail can ensure self-renewal proliferation of human HBs that holds a great potential for HB transplantation mediated repair of liver injuries.

## Supplementary information

**Additional file 1.** Supporting information.

## Data Availability

The datasets supporting the conclusions of this article are included within the article.

## Abbreviations

hPSCs: Human pluripotent stem cells
iPSCs: Induce pluripotent stem cells
ESCs: Embryonic stem cells
HBs: Hepatoblasts
HH: Hedgehog
DE: Definitive endoderm
HE: Hepatic endoderm
HLCs: Hepatocyte-like cells
PH: Primary hepatocyte
FACS: Fluorescence-activated cell sorting
NSI mice: NOD-SCID-IL2RG^−/−^ mice
CHIR: CHIR99021
DM: Dorsomorphin
VM: Vismodegib
SB: SB431542
FSK: Forskolin
CYP: Cytochrome P450
