# Supplementary material for: Synergistic modulation of signaling pathways to expand and maintain the bipotency of human hepatoblasts
Source: Stem Cell Res Ther. 2019 Dec 2;10:364. doi: 10.1186/s13287-019-1463-y (PMC6888929; doi:10.1186/s13287-019-1463-y)
Supplement: Supplementary file 1 — Additional file 1. Supporting information. [file 13287_2019_1463_MOESM1_ESM.docx]

**Additional file 1**

**Synergistic modulation of signaling pathways to expand and maintain the bipotency of human hepatoblasts**

Tingcai Pan^1,2,3,4,§^, Yan Chen^1,3,4,§^, Yuanqi Zhuang^1,3,4^, Fan Yang^1,3,4^, Yingying Xu^1,3,4^, Jiawang Tao^1,2,3,4^, Kai You^1,3,4^, Ning Wang^1,3,4^, Yuhang Wu^1,3,4^, Xianhua Lin^1,3,4^, Feima Wu^6^, Yanli Liu^6^, Yingrui Li^7^, Guodong Wang^8^ and Yinxiong Li^1, 2, 3, 4, 5,^ *

^1^ Institute of Public Health, Guangzhou Institutes of Biomedicine and Health, Chinese Academy of Sciences, Guangzhou, 510530, China

^2^ University of Chinese Academy of Science, Beijing, 100049, China

^3^ Key Laboratory of Regenerative Biology, South China Institute for Stem Cell Biology and Regenerative Medicine, Guangzhou Institutes of Biomedicine and Health, Chinese Academy of Sciences, Guangzhou, 510530, China

^4^ Guangdong Provincial Key Laboratory of Biocomputing, Guangzhou Institutes of Biomedicine and Health, Chinese Academy of Sciences, Guangzhou, 510530, China

^5^ Guangzhou Regenerative Medicine and Health Guangdong Laboratory, 510005 Guangzhou, China

^6^ The Second Affiliated Hospital, Guangzhou Medical College, Guangzhou 510260, China

^7^ iCarbonX(Shenzhen) Company Limited, Shenzhen, 518000, China

^8^ The First Affiliated Hospital, Sun Yat-Sen University, Guangzhou, 510080, China

^§^ Authors contributed equally to this study as co-first authors

*Correspondence: li_yinxiong_iph@gibh.ac.cn

**Additional file 1: Figures**


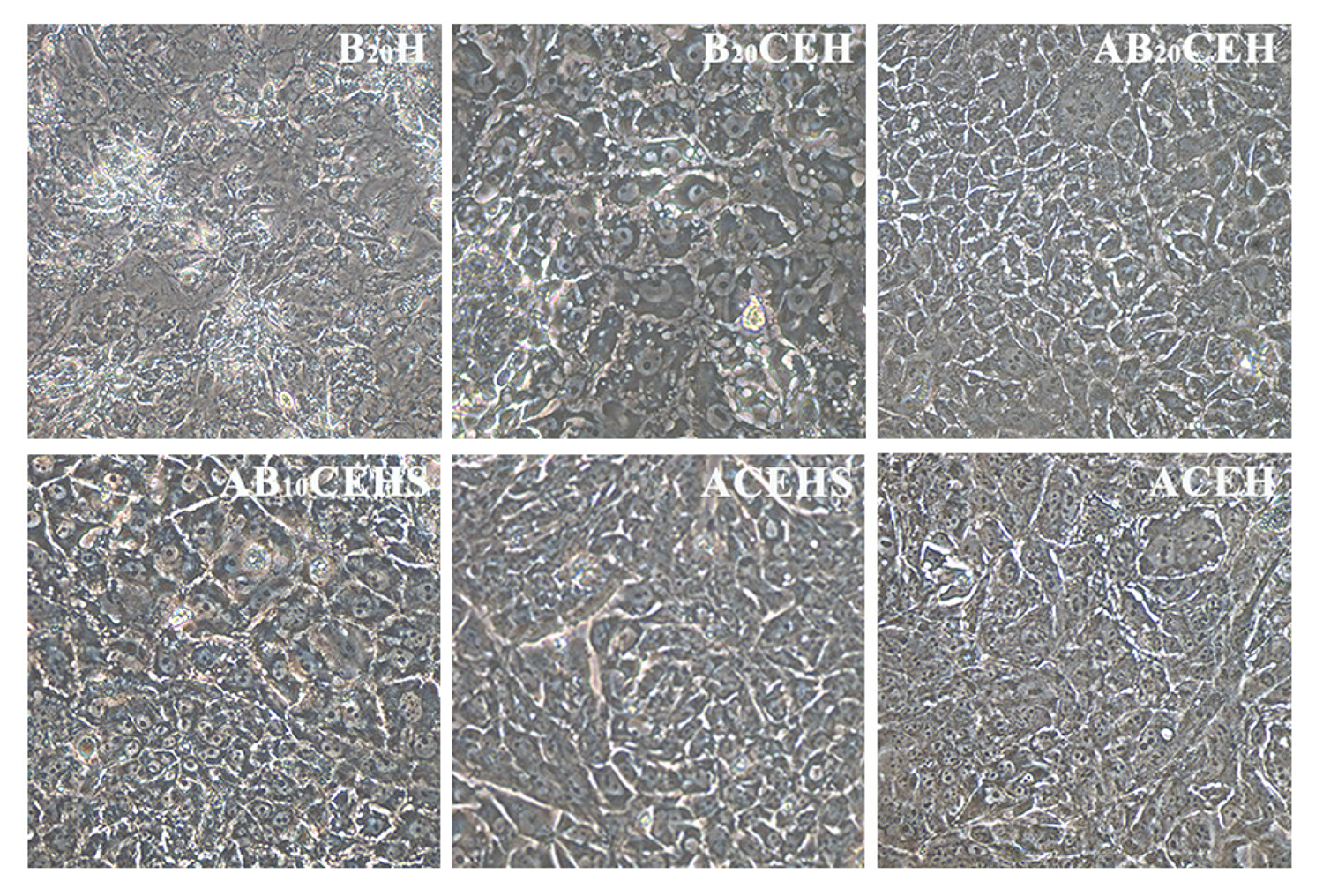


**Fig. S1 Morphologies of expanding HBs under different culture conditions as indicated in each panel.**

The basic B_20_H cocktail had minimal proliferation activity and the majority of cells lost their epithelial shape shifting to a fibroblastic shape. Addition of CHIR and other growth factors resulted in increased the proliferative ability.


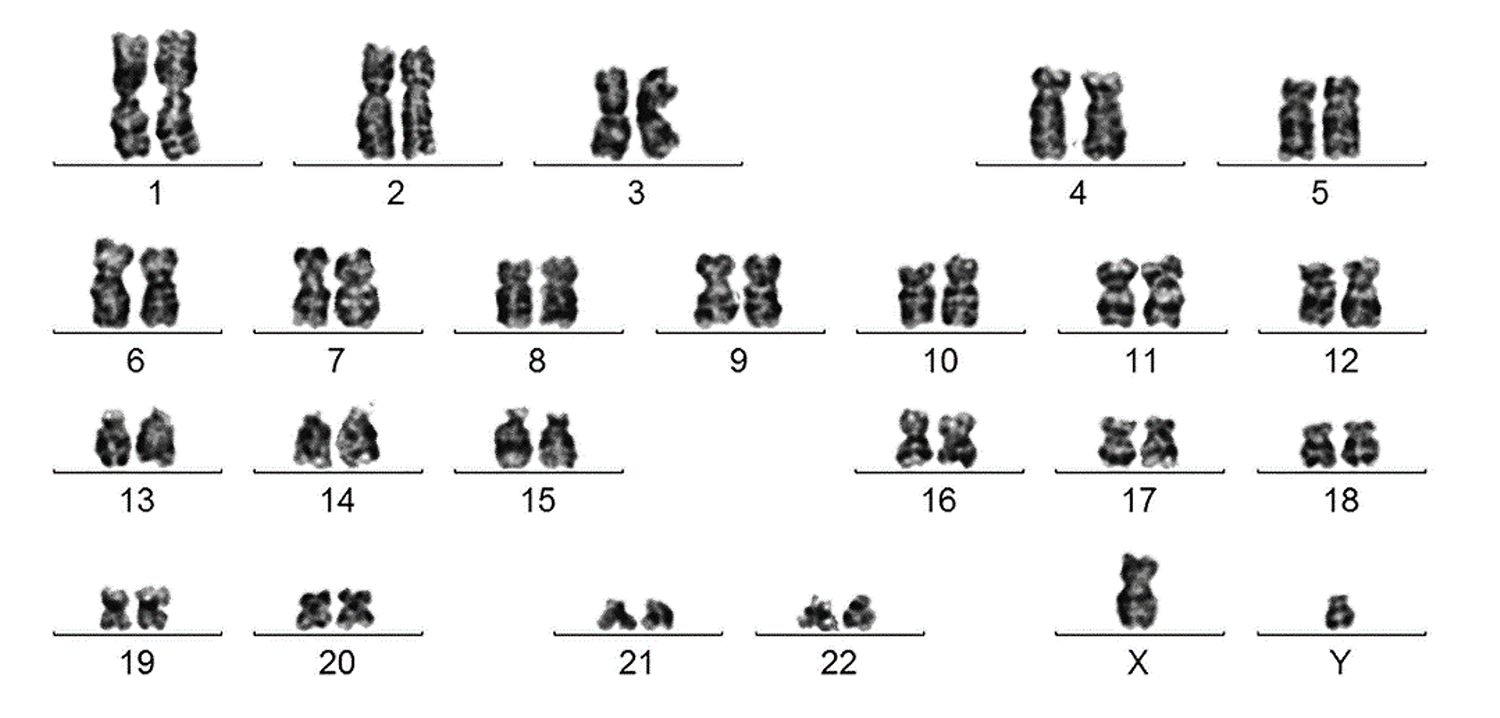


**Fig. S2 The expanded HBs showed normal karyotype.**

The expanded HBs retained normal karyotypes after long-term culture (10 passages).


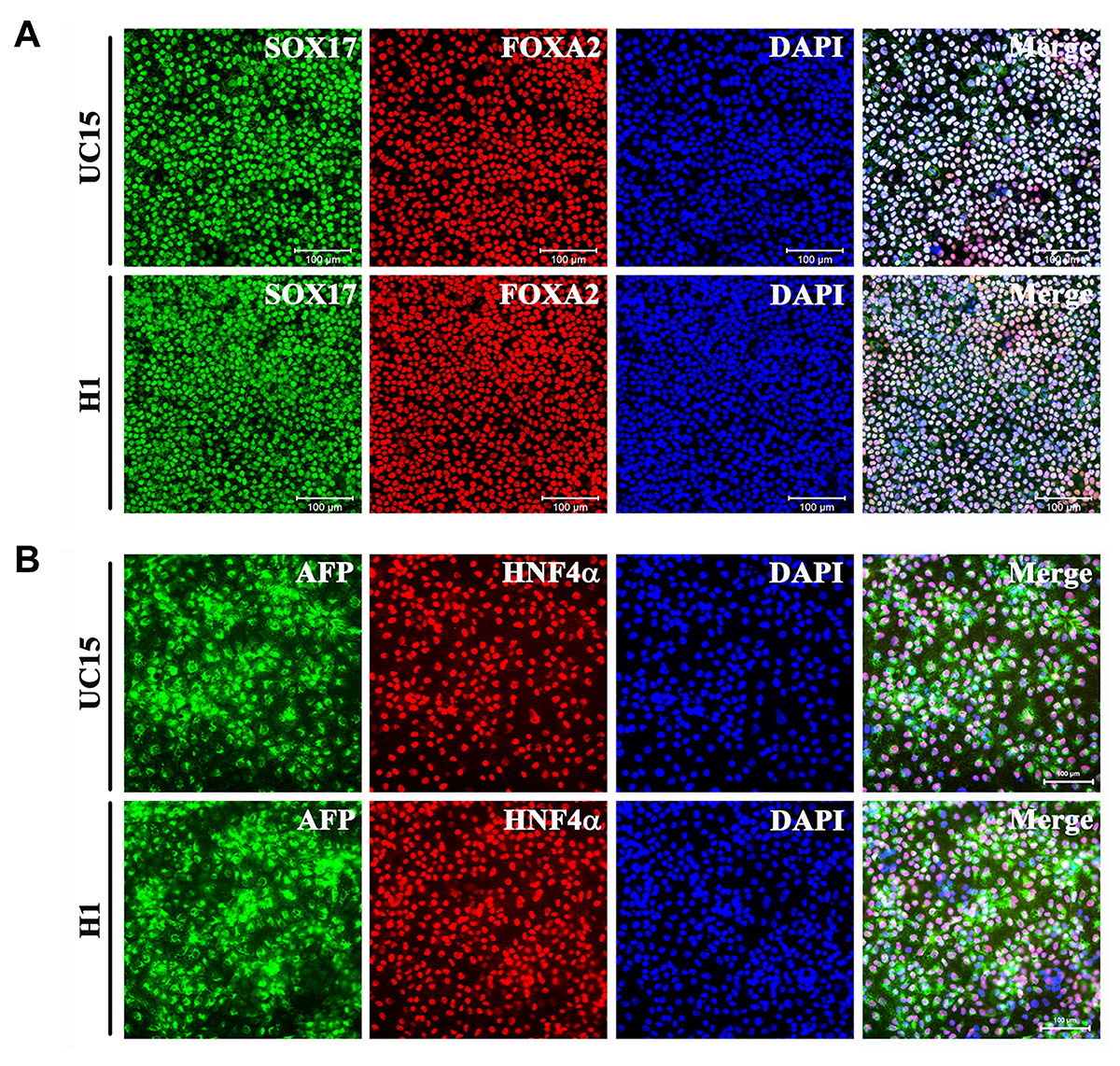


**Fig. S3 Generation of HBs from other human iPSC line and ESC line.**

Immunostaining analyses results showed that human iPSC line (UC15) and ESC line (H1) also could sequentially differentiate into DE cells (A) and HBs (B) with high efficiency similarity as previous UC01 cells, and expressed stages specific markers.


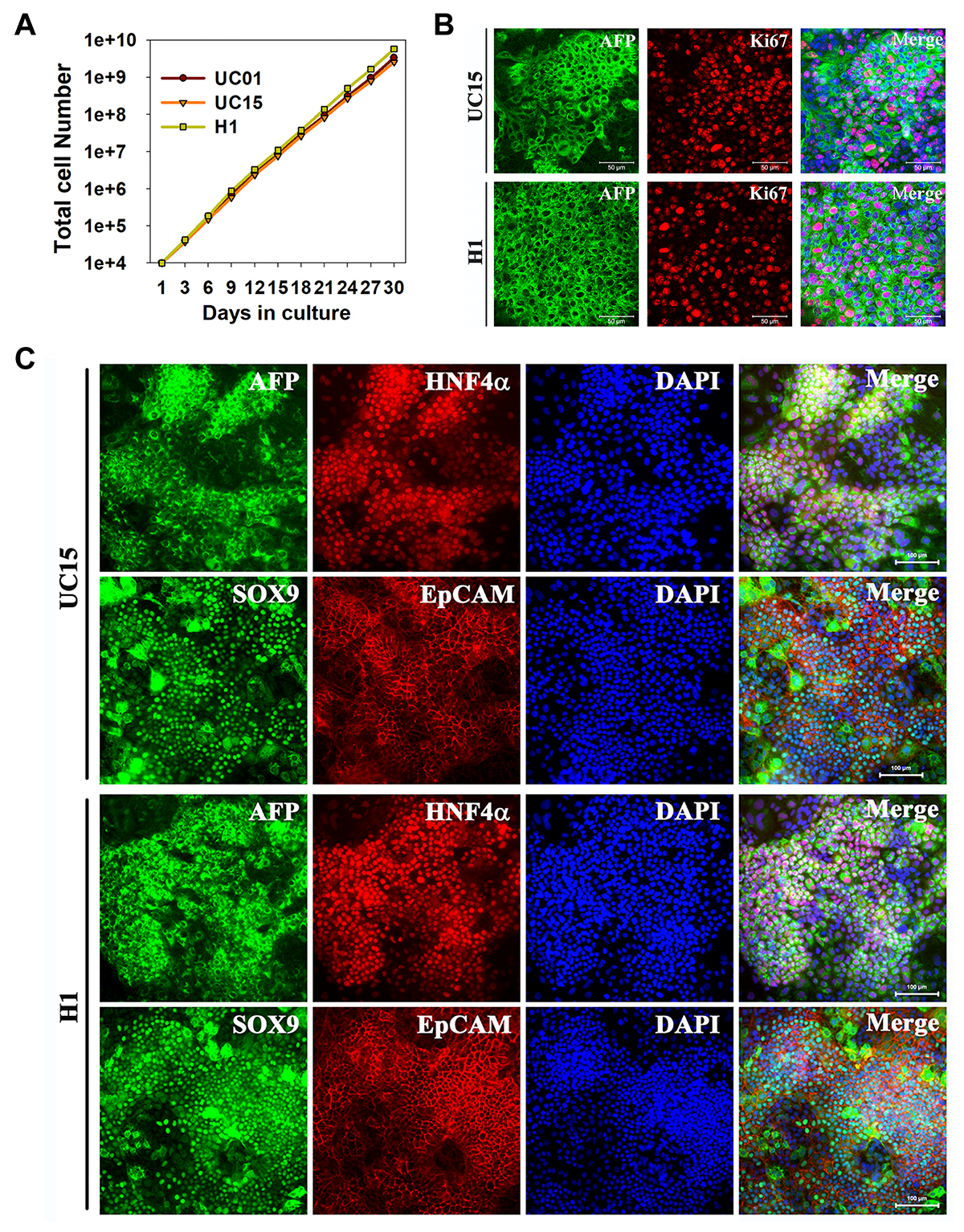


**Fig. S4 Expansion and characteristics analyses of the expanded HB derived from other human iPSCs or H1 ESCs.**

(A) HBs derived from human iPSCs or H1 ESCs were cultured in optimized condition (AB_10_CEHS) for 30 days (10 passages) expansion, and cell growth was analyzed by cell count. (B) AFP and Ki67 expression was analyzed by immunostaining on expanded HBs that after 30 days (10 passages) expansion. (C) Immunostaining analyses of HBs after 30 days (10 passages) expansion.


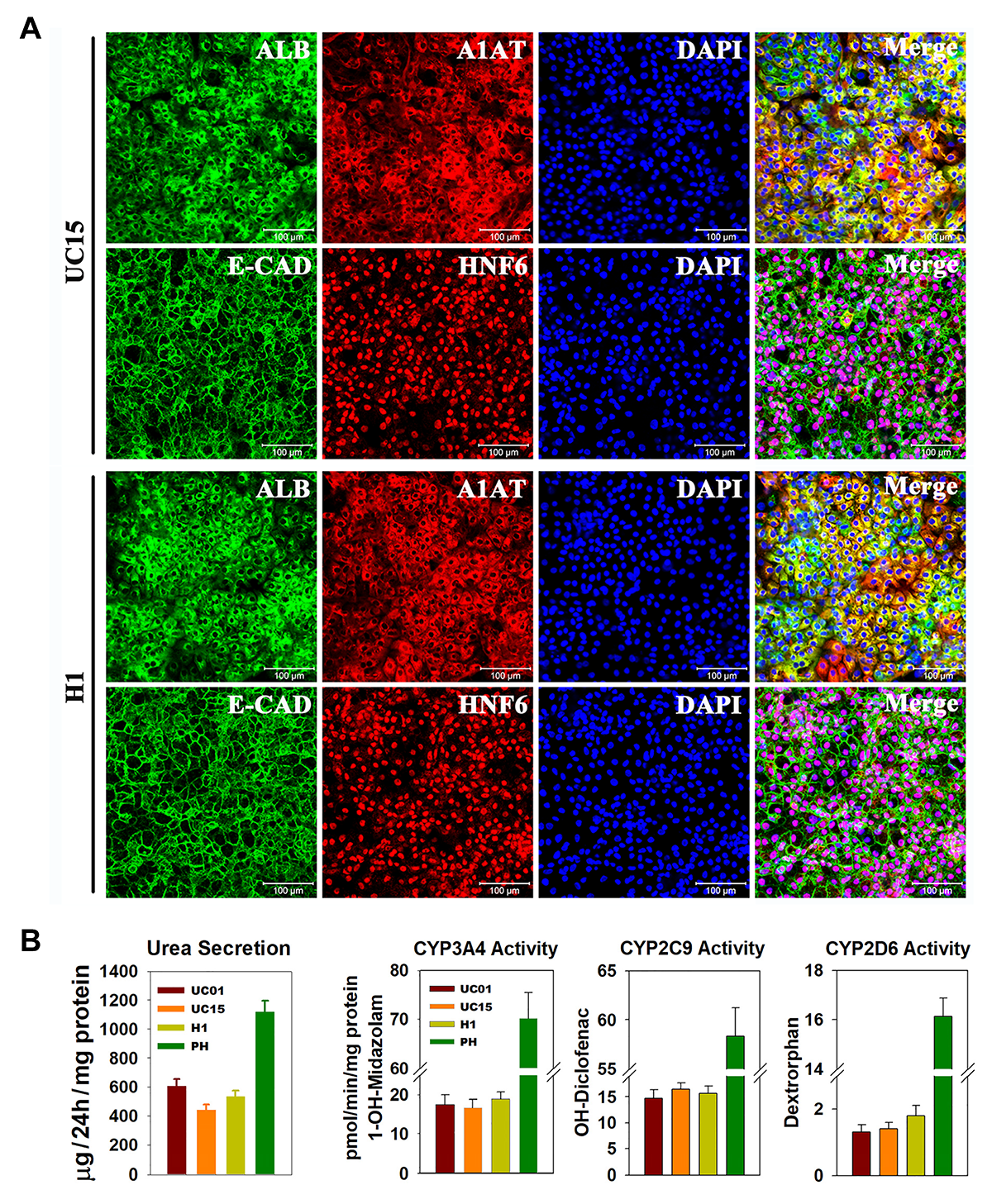


**Fig. S5 Differentiation of expanded HBs into functional hepatocytes.**

Expanded HBs (10 passages) derived from human iPSCs or H1 ESCs were differentiated into hepatocytes, and harvested for analyses. (A) Hepatocytes derived from two cell lines (UC15 or H1) both express mature hepatocyte-specific markers, including ALB, A1AT, E-CAD and HNF6. (B) Urea secretion and CYP450 activity assay of different origin of hepatocytes. Data are presented as mean ± SEM, n = 3.


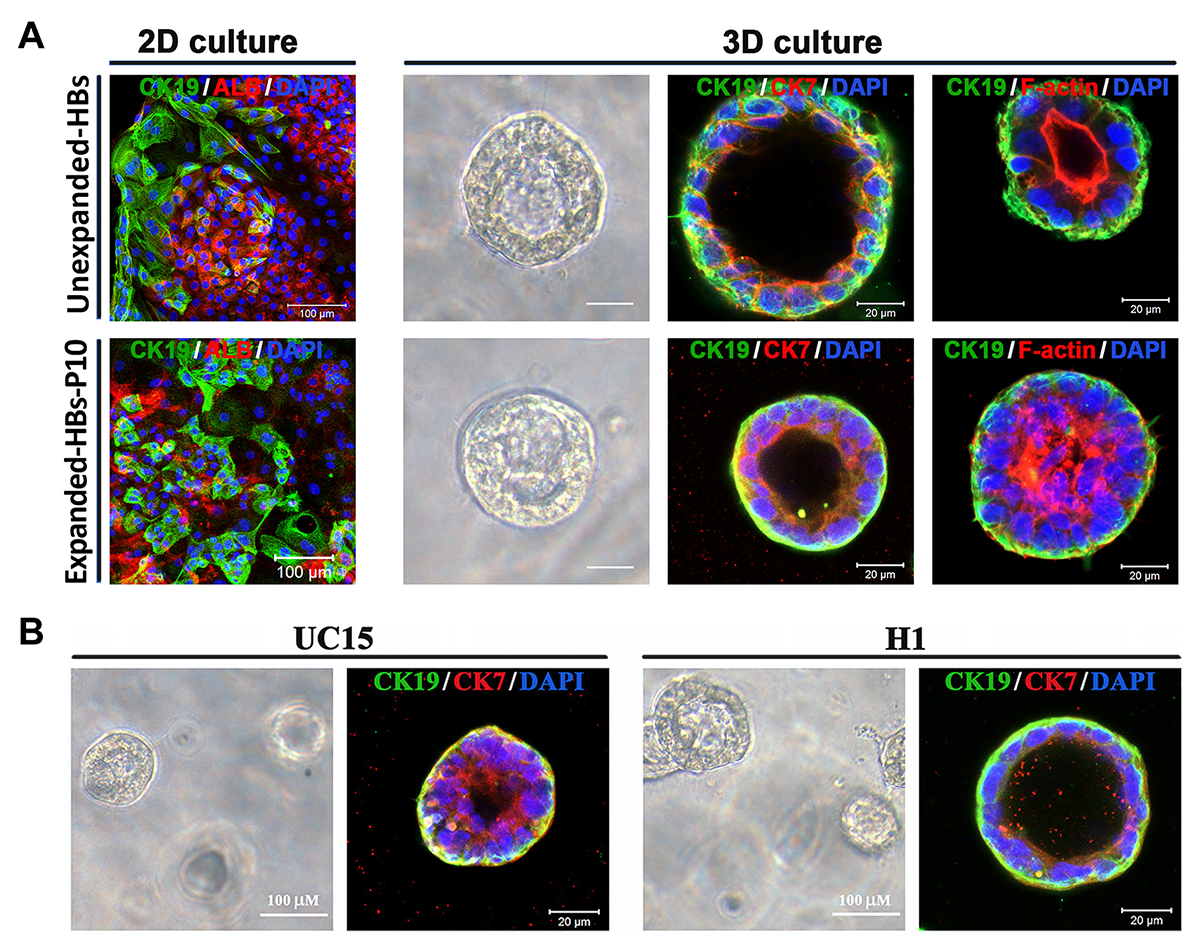


**Fig. S6 Differentiation of HBs into cholangiocyte-like cells and bile duct-like structures.**

After 7 days induction, (A, Left) HBs could differentiate into CK19-positive cholangiocyte-like cells in monolayer culture. (A, Right) While in 3D culture condition, HBs form bile duct-like structures, and demonstrated epithelial polarity with CK7 and CK19 on the basolateral region and F-actin at the apical region. (B) Expanded HBs derived from UC15 or H1 also could differentiate into CK19 and CK7 positive bile duct-like structures in 3D culture.

**Tables**

**Table S1. List of antibodies used in this study**

| Primary anbibody | Species | Company | Cat Number | Dilution |
| --- | --- | --- | --- | --- |
| OCT4 | Mouse | Genetex | GTX627423 | 1:200 |
| Nanog | Rabbit | Genetex | GTX100863 | 1:200 |
| FOXA2 | Goat | R&D systems | AF2400 | 1:200 |
| SOX17 | Mouse | Genetex | GTX83580 | 1:100 |
|  | Goat | R&D systems | IC1924A | 1:50 |
| C-kit | Mouse | Thermo Fisher | 17-1178-42 | 1:100 |
| EpCAM | Mouse | Invitrogen | 53-8326-42 | 1:100 |
|  | Mouse | Abcam | ab46714 | 1:100 |
| ALB | Goat | R&D systems | MAB1455 | 1:200 |
|  | Rabbit | Dako | F0117 | 1:50 |
| HNF4α | Rabbit | Genetex | GTX62347 | 1:200 |
| AFP | Mouse | Genetex | GTX84948 | 1:200 |
| Ki67 | Rat | Thermo Fisher | 11-5698-82 | 1:100 |
|  | Rabbit | Genetex | GTX16667 | 1:200 |
| E-CAD | Goat | R&D systems | AF648 | 1:200 |
| CYP3A4 | Mouse | Genetex | GTX60577 | 1:200 |
| HNF6 | Mouse | Genetex | GTX83966 | 1:200 |
| CK19 | Mouse | R&D systems | AF3506 | 1:200 |
| CK7 | Rabbit | Genetex | GTX109723 | 1:200 |
| SOX9 | Mouse | Abcam | ab76997 | 1:200 |

| Second antibody | Species | Company | Cat. Number | Dilution |
| --- | --- | --- | --- | --- |
| Anti-Goat IgG Antibody, Alexa Fluor 488 | Donkey | Invitrogen | A-11055 | 1:500 |
| Anti-Goat IgG Antibody, Alexa Fluor 568 | Donkey | Invitrogen | A-11057 | 1:500 |
| Anti-Mouse IgG Antibody, Alexa Fluor 488 | Donkey | Invitrogen | A32766 | 1:500 |
| Anti-Mouse IgG Antibody, Alexa Fluor 568 | Donkey | Invitrogen | A10037 | 1:500 |
| Anti-Rabbit IgG antibody Alexa Fluor 488 | Donkey | Invitrogen | A-21206 | 1:500 |
| Anti-Rabbit IgG antibody Alexa Fluor 568 | Donkey | Invitrogen | A10042 | 1:500 |

**Table S2. List of primers for real-time PCR**

| **Gene** | **Primer sequence (forward / reverse; 5' to 3')** |
| --- | --- |
| *AFP* | AGAACCTGTCACAAGCTGTG / GACAGCAAGCTGAGGATGTC |
| *HNF4α* | TGTACTCCTGCAGATTTAGCC / CTGTCCTCATAGCTTGACCT |
| *ALB* | TGGCACAATGAAGTGGGTAA / CTGAGCAAAGGCAATCAACA |
| *CK19* | GAACCATGAGGAGGAAATCAG / CATGTCACTCAGGATCTTGG |
| *PDX1* | ACCAAAGCTCACGCGTGGAAA / TGATGTGTCTCTCGGTCAAGTT |
| *CDX2* | GGGCTCTCTGAGAGGCAGGT / GGTGACGGTGGGGTTTAGCA |
| *SOX17* | GTGGACCGCACGGAATTTG / GGAGATTCACACCGGAGTCA |
| *SHH* | ACCGAGGGCTGGGACGAAGA / ATTTGGCCGCCACCGAGTT |
| *IHH* | CATTGAGACTTGACTGGGCAAC / AGAGCAGGCTGAGTTGGGAGTCGC |
| *PATCH* | CCACCAGACGCTGTTTAGTCA / CGATGGAGTCCTTGCCTACAA |
| *Tgf-β1* | CTAATGGTGGAAACCCACAACG / TATCGCCAGGAATTGTTGCTG |
| *Tgf-β2* | CCCCGGAGGTGATTTCCATC / GGGCGGCATGTCTATTTTGTAAA |
| *Tgf-β3* | ACTTGCACCACCTTGGACTTC / GGTCATCACCGTTGGCTCA |
| *Wnt3* | GCCCCACTCGGATACTTCTTACT / GAGGAATACTGTGGCCCAACA |
| *Axin2* | CCCCAAAGCAGCGGTGC / GCGTGGACACCTGCCAG |
| *c-Myc* | GCTCCGTTTTACCTCGTGCC / GCTCCGTTTTACCTCGTGCC |
| *Ki67* | GCCTGCTCGACCCTACAGA / GCTTGTCAACTGCGGTTGC |
| *GAPDH* | CAAAGTTGTCATGGATGACC / CCATGGAGAAGGCTGGGG |

**Supplemental Experimental Procedures**

**Quantitative RT-PCR and microarray analysis**

Total RNA was extracted using TRIzol reagent (Invitrogen) according to the manufacturer’s protocol and quantified with NanoDrop 2000 (Thermo Fisher). cDNA was reverse transcribed from 2 μg RNA using ReverTra Ace (Toyobo) and oligo-dT (Takara). Quantitative RT-PCR was performed with CFX96 machine (Bio-Rad) and SYBR Green Premix (Bio-Rad) following the manufactures’ manual. The GAPDH was used for quantitative RT-PCR normalization, and the experiments were repeated a minimum of three times to confirm the results. Primer sequences are listed in the Supporting information Table 2.

**RNA sequencing**

For RNA sequencing, total RNA was prepared from iPSCs, unexpanded (P0) and expanded (P10 and P20) HBs which derived from different colonies. About 4 μg of total RNA was used to generate sequencing-ready cDNA library with the TruSeq RNA Sample Prep Kit (Illumina, RS-122-2001). Each library was sequenced using single-reads in HiSeq2000/1000 (Illumina). Gene expression levels were analyzed using Cufflinks.

**Immunohistochemistry staining**

Cells were washed with PBS and fixed with 4% PFA (Sigma-Aldrich) for 30 min at room temperature. After washing the cells with PBS, cells were permeabilized with 0.1% Triton X-100 in PBS for 30 minutes, and blocked with PBS containing 5% normal goat or donkey serum for 30 minutes at room temperature. The primary antibodies diluted with blocking solution and incubated at 4°C for overnight. After washing the cells with PBS, cells were then stained with compatible Alexa Fluor-conjugated secondary antibodies in blocking solution for 1h at room temperature. Nucleus were stained with 5 μg/mL DAPI (Invitrogen). Imaging was performed on a Zeiss LSM 710 confocal microscope. The primary antibodies and secondary antibodies described in the Supporting information Table 1.

**Fluorescence-activated cell sorting (FACS) and flow cytometry analysis**

Cells were dissociated with Accutase and then resuspended in PBS containing 3% BSA. The collected cell suspensions stained with FITC-conjugated human Ep-CAM antibody (Milteny) and Alexa Fluor APC-conjugated antibody against human C-Kit (B56; BD Biosciences) for 30 minutes on ice. EpCAM^+^ and C-Kit^-^ cells were sorted using a MoFloTM fluorescence-activated cell sorter. Sorted cells were seeded on Matrigel pre-coated plates, and maintained in expansion basal medium containing growth factor and small molecule cocktail. Colony forming activities of these cells were analyzed.

To analyze the proliferation of hepatic cells, cells were dissociated with Accutase and then resuspended in PBS containing 10% FBS. The collected cell suspensions were fixed with 4% PFA (Sigma-Aldrich), and incubated in blocking and permeabilizing buffer, containing 0.1% Triton X-100, and 5% normal donkey serum in PBS for 30 minutes at room temperature. The cells were then incubated with APC-conjugated Ki67 (B56; BD Biosciences) and other indicated antibody for 30 minutes on ice. Corresponding isotype antibodies were used as controls. Flow cytometry analyses were performed using a FACS Aria II flow cytometer (BD Biosciences).

**Karyotype analysis**

Cells were cultured in a 60-mm dish until 80% confluence, and then treated with colcemid (0.1 g/mL, final concentration) for 2 hours before harvesting. After washed with PBS, cells were then dissociated with Accutase, transferred into 15-mL tubes, and centrifuged with the supernatant removed and resuspended with 10 mL KCl solution (75 mM). The cell mixtures were then incubated for 30 minutes in 37°C water bath and then fixed by adding 1 mL fresh fixative solution (methanol/acetic acid, 3:1). The fixed cells were washed at least 3 times with 10 mL of fixative solution before being applied onto chilled slides. The slides were air-dried, stained with DAPI, and observed under a fluorescent microscope. At least 40 fields with clearly spread chromosomes were examined.
